# Research-related knowledge, understanding and practice in public mental health: the voices of social workers and occupational therapists

**DOI:** 10.1186/s12961-024-01195-7

**Published:** 2024-08-05

**Authors:** Christine Migliorini, Megan Turville, Caitlin McDowell, JoAnne Bevilacqua, Carol Harvey

**Affiliations:** 1 Division of Mental Health, North West Area Mental Health, Northern Health, Melbourne, VIC Australia; 2 Psychosocial Research Centre, Department of Psychiatry, University of Melbourne, Melbourne. VIC, Australia; 3 Centre for Youth Mental Health, University of Melbourne, Melbourne, VIC Australia; 4 Department of Psychiatry, University of Melbourne, Melbourne, VIC Australia

**Keywords:** Allied health, Research capacity building, Research culture, Workforce development, Mental health, Adult

## Abstract

**Introduction:**

Previous studies have explored facilitators and barriers to research conducted by allied health professionals in general medical settings. Since the mental health system is acknowledged to be significantly under-funded and more poorly functioning than general medical services, it is unclear whether the published facilitators and barriers also apply to mental health settings. This study sought to explore the research-related knowledge, understanding and practices of allied mental health clinicians based in a large public mental health service.

**Methods:**

A mixed methods study recruited 59 occupational therapists and social workers working in a dedicated metropolitan public mental health service in Melbourne, Australia. Quantitative survey results are reported elsewhere. Semi-structured interviews were conducted with 16 survey responder volunteers. Thematic analysis was conducted on the qualitative survey and interview data.

**Results:**

Four main themes were identified: research must connect with clinical practice; fragments of knowledge; research in practice; and research is not part of my professional identity. The third theme, research in practice, comprised four subthemes: no time for research in clinical roles, missing communication, lack of ownership, and what I need to do research.

**Conclusions:**

This study found that research and research-related activities were not considered part of the mental health social workers and occupational therapists’ professional identities. Dealing with this issue may be instrumental to the realization of these clinicians’ professional peak-body associations’ code of practice and to government mandated practice standards. We provided several strategies to encourage both clinicians and services to view research-related activities as an everyday part of clinical roles. This is especially important if we think of allied health evidence-based practice requiring a reasonable level of research-related skills and/or competencies to appraise, practice, evaluate and adapt their evidence-based practice.

## Introduction

Allied health professionals, including occupational therapists and social workers, are under increasing pressure to deliver evidence-based practice (EBP) or evidence-supported interventions in a timely and cost-effective manner. It is both expected and ratified in their discipline’s codes of practice (e.g. see [1, 2]). EBP is mandated by government health department guidelines and practice frameworks (e.g. AHPRA Code of Practice, Principle 1 [3]). It is also demanded by patients, consumers and carers. The importance cannot be overestimated.

The evidence in EBP is accrued and evaluated through research and research methodology. What do we mean by research? According to the NHMRC Australian National Statement on Ethical Conduct in Human Research, “human research is any investigation that is conducted with or about people, or their data or tissue” (p. 7) [4]. Research is about acquiring knowledge and uncovering causes and solutions by identifying a problem and then systematically collecting and appraising information about the problem and developing explanations, interventions and policy improvements (adapted from Bordens & Abbot 1999, p. 3) [5]. Skills and knowledge are also needed to judge the utility and generalizability of published interventions, as well as applicability to one’s own practice and evaluation of same. Accordingly, research-related skills and knowledge are important to the delivery of EBP [6].

According to the Victorian Allied Health Research Framework, all allied health practitioners have a professional responsibility to engage with research, with the capacity to understand and apply evidence [7]. Research is also highlighted in various allied health disciplines’ practice standards. For example, the Australian Social Work Association [8] advises that: social workers should have the skills and knowledge to generate new knowledge for practice, including proposing innovative research; having appropriate knowledge of research methodologies; distinguishing and evaluating various sources of knowledge including, in part, research evidence, and disseminating research knowledge. Occupational Therapy Australia [9] advises that it is the responsibility of the discipline to generate new research, apply research in practice and critically evaluate the quality of evidence.

Several studies have surveyed health practitioners about the barriers and motivators to conducting research. Motivators tended to fall within the two themes of personal interests and practice-based interests. Personal interest motivators included increasing job satisfaction, developing their skills, keeping their brains stimulated, career advancement and personal drive [10–16]. Practice-based motivators centred on identification of problems requiring change and improving clinical care [10, 12–17]. Barriers tended to fall within the two themes of personal interests and organization context/resources. Personal-interest-related barriers included lack of (or limited) skill, desire for work/life balance, perceived complexity of research and fear of getting it wrong and previous bad experience with university supervisor [10, 11, 13, 14, 16, 18, 19]. Interestingly, in one study approximately a third of participants were unsure whether research was a requirement of their role [18]. Lack of time, other work priorities and lack of funding [10–18, 20–22] dominated the reported organization context/resource themed barriers, but those barriers also included lack of suitable backfill, administrative support and availability of technical expertise [11, 12, 14, 16, 18].

Both motivators and barriers are important to note. However, those studies were based in general medical settings. There is growing evidence that mental health settings may be under more intense, widespread pressure as they try to function in a broken system with comparatively less funding. Public mental health services have been a poor cousin in the receipt of health funding in Australia for some time. For example, only 7.6% of government health expenditure was spent on mental-health-related services in 2019–2020, consistent with that for 2015–2016 (7.6%) [23]. While mental illness and substance abuse disorders contributed to 15% of Australia’s total burden of disease in 2023 [24], suggesting that funding would need to roughly double to make it reflective of the impact of mental illness [25]. It should come as no surprise then that in 2020, the Australian Productivity Commission found that Australia’s current mental health system was not comprehensive and needed to be reformed [26]. Considering the relatively poor funding and functioning of mental health services, it seems plausible that some of the organization context/resource-themed barriers will be further amplified and that less emphasis might be placed on research being a core component of practitioners’ roles. Consequently, it is unclear whether the published literature will straightforwardly translate to mental health settings or whether there may be elements unique to mental health settings.

A further consideration is the use of the term research. At the beginning of Kothari’s research methodology book [27], several definitions were offered, ranging from the simple “search for knowledge” to “an original contribution to the existing stock of knowledge making for advancement” and “systematic method consisting of enunciating the problem, formulating a hypothesis, collecting the facts or data, analysing the facts and reaching certain conclusions” (pp. 1–2). NHMRC acknowledges that at present there is no formally agreed upon definition of research [4]. Many studies explore research without providing a clear definition, leaving the interpretation to the participant. It is unclear whether academic researchers and mental health professionals/clinicians think of research in the same way. Therefore, this study approached allied mental health clinicians based in a large public mental health setting to explore their research-related knowledge, understanding and practice. This focus included finding out what clinicians think research is and how it is undertaken.

## Methods

An exploratory mixed methods study design, consisting of an online survey and semi-structured interviews conducted via web-based teleconference, was implemented. The anonymous survey, the results of which have been reported elsewhere [28], concluded with a call for volunteers willing to be interviewed. The current study will focus on responses to the open-ended survey questions and transcripts from the semi-structured interviews. Survey data were collected within a 6-week period in October and November 2020, and managed using REDCap [29], an electronic data capture tool hosted by the Royal Melbourne Hospital Business Intelligence Unit. The interviews were conducted concurrently.

### Research team description

Our research team consisted of four PhD-qualified researchers experienced in both qualitative and quantitative research and an additional team member with limited research experience but extensive service delivery experience. All team members had mental-health-related post-graduate qualifications, covering the disciplines of mental health social work, occupational therapy and psychiatry. More than 70 years of experience working with individuals with mental illness was embodied by the research team. Role responsibilities of the team members included training and supporting clinicians in their research-related activities.

### Workplace setting

The study was set within a large metropolitan public mental health service based in Victoria, Australia. The organization provides a comprehensive range of specialist-, community- and hospital-based mental health services for youth, adult and aged people who are experiencing, or at risk of developing, a severe mental illness. Services are delivered across a range of locations, including most major hospitals within the region, and a number of community-based clinics. At the time of the study, it also included services that delivered several state-wide specialist services encompassing neuropsychology and eating disorders. Our study focussed on social workers and occupational therapists. Arguably these two allied health disciplines receive very similar levels of research-related training during their undergraduate years.

### Recruitment method

All social workers and occupational therapists employed within the service at the time of the study received a recruitment email with an invitation to participate in the study, an attached plain language statement and a link to the anonymous online survey via their “discipline-specific all-staff” emailing list. The recruitment email was re-sent on a further two occasions within the 6-week period in October and November 2020. The inclusion criteria for the study were being an academically qualified social worker or occupational therapist employed by the service as mental health clinicians at the time of recruitment, regardless of position/role, hours worked per week or whether employed on a full-time, part-time, contract or casual basis. No financial reimbursement was offered. The organization approved the completion of the survey and interview (if they volunteered) during participants’ work time, should they wish.

### Online survey

The online survey comprised three broad sections. Section 1 canvassed background information including demographic, discipline-related characteristics and the range and type of research-related activities with which participants had been involved over the previous 5 years, providing the following options: project work, quality assurance, evaluation and research. This section then finished with the following single open-ended question: In your view, what are the differences, if any, between these terms? Section 2 comprised multiple choice questions, including the validated Research Capacity and Culture tool, fully described and with results reported elsewhere [28]. Please note that section 2 began on a separate page and opened with the statement: For the purposes of the remaining survey questions, quality assurance, evaluation and research will be referred to as simply “research”. Section 3 comprised the following open-ended questions:

- *Please tell us about any specific training that you have completed that supports your research-related activities.*
- *What sort of resources have you used to support your research-related activities (internal and/or external)?*
- *Are you interested in pursuing research-related activities yourself? And what sort of things would you like to do?*
- *What has been the toughest research-related task that you have undertaken? Please describe.*
- *What type of research-related tasks do you see others struggling with the most? Please describe.*
- *What would be the best way of supporting your research-related activities? Please describe.*
- *On reflection, what is one thing we could do to help you undertake research-related tasks at [this mental health service]? Please describe.*

### Interviews

The survey’s open-ended questions also underpinned the four topics of the interview guide: (a) recent experiences of research process; (b) explore their understandings about research and any research-related training; (c) opportunities for improvement; and (d) future priorities/direction. (See supplemental material for example probing questions associated with each topic). The online interviews were chosen to augment the survey since open-ended survey questions typically do not receive comprehensive detailed responses [30]. Thus, interviews with a subset of survey completers enabled the researchers to explore survey themes in more depth. The interviews were conducted largely within the same 6-week period via Zoom Video Communications software. The interview volunteers had opted in at the relevant section of the survey. On receipt of a completed survey where the participant had opted to be interviewed, a research team member contacted the volunteer to make the interview appointment and then conducted the online interview at a mutually agreed upon time. Three members of the research team conducted the interviews (C.M., C.M.c.D., J.B.). The research team ensured that each interview was conducted in private by a team member with no formal connection with the interviewee, that is, different discipline background and never worked within the same team.

### Analysis and rigour

An experiential thematic analysis located within a phenomenological paradigm [31, 32] was undertaken to examine the written survey responses and interview transcripts, extracting meaningful units of information that focus on participants’ knowledge, understanding and practice, which was a key focus of this study. Data from the open-ended survey questions and the interview transcripts were consistent and therefore were analysed and reported together.

MS Excel for MS365 v2302 was used to summarize the demographic data and analyse both the qualitative survey responses and professionally transcribed interview data. SPSS v29 was used for the chi-squared test for independence (with Yates continuity correction where applicable) analyses, comparing demographic characteristics of participants who completed the survey only with those of survey responders who also volunteered to be interviewed. *P*-values less than 0.05 were deemed statistically significant.

A research team consensus-building framework guided our approach. Research team members (C.M. and M.T.) separately coded the transcripts and qualitative survey responses, coming together intermittently to discuss and reach consensus of emergent themes. Once this stage was completed, the themes were taken to the broader research team for further discussion of connections across the emergent themes until consensus of themes was reached, as described by Braun and Clark [31]. Corroboration brought about by the dual collection methods (survey and interview) help build credibility and confidence in the study findings. This could be framed as methodological triangulation by using different methods for collecting the data, thus providing a richer understanding of the phenomena being studied [31, 33]. While the sampling timeframe was dictated by time commitment/budgetary considerations, no new information was being collected by the end of the sampling timeframe, suggesting the point of redundancy had been reached [32].

### Ethics

All applicable institutional and governmental regulations concerning the ethical use of human volunteers and in accordance with the 1964 Declaration of Helsinki and its later amendments were followed during this research. Each participant received a plain language statement and opportunity to ask questions about the study before completing the survey, and when applicable, commencing the interview. Informed consent was deemed given with the voluntary completion of the survey. Each interviewee had volunteered to be interviewed, with verbal consent recorded at the beginning of the interview. This project received full ethics approval from the Melbourne Health Ethics Committee: HREC/6418/MH-2020.

## Results

### Participant demographics

In total, 59 mental health clinicians completed the online survey. A little over half of the participants were social workers. Most participants identified as female , and the modal average age group was 35–49 years. Postgraduate coursework was the most common education level either attained or currently being undertaken , and participants tended to be either less than 5 years since completing their last qualification or more than 10 years post-qualification. Many participants did have some research-related experience while nearly a third of the sample did not. Sixteen  (survey volunteer) clinicians were also interviewed. Mean average interview length was 32 min (SD = 5 min). Comparative statistical analyses showed that participants who were interviewed did not differ from individuals not interviewed according to discipline, gender, age group, level of education, time since qualification or research experience. See Table 1.

**Table 1:** Demographic, discipline and research activity characteristics of survey and interview participant comparisons

Variable	Sub-category	Survey participants *not* interviewed	Survey participants interviewed	*p*-value
		Count	Count	
Discipline				
Social worker		24	11	0.55
Occupational therapist		19	5	
Gender				
Female		38	11	0.16
Male		5	5	
Other		–	–	
Age				
20–34 years		15	3	0.33
35–49 years		18	7	
50+ years		9	6	
Highest education				
Undergraduate only		13	4	0.29
Post-graduate coursework (completed or ongoing)		26	8	
Post-graduate research (completed or ongoing)		4	4	
Time since last qualification				
< 5 years		19	3	0.20
5–10 years		10	5	
11+ years		14	8	
Has research-related activity experience				
Yes		13	6	0.83
No		30	10	

### Thematic analysis

**Fig. 1:**
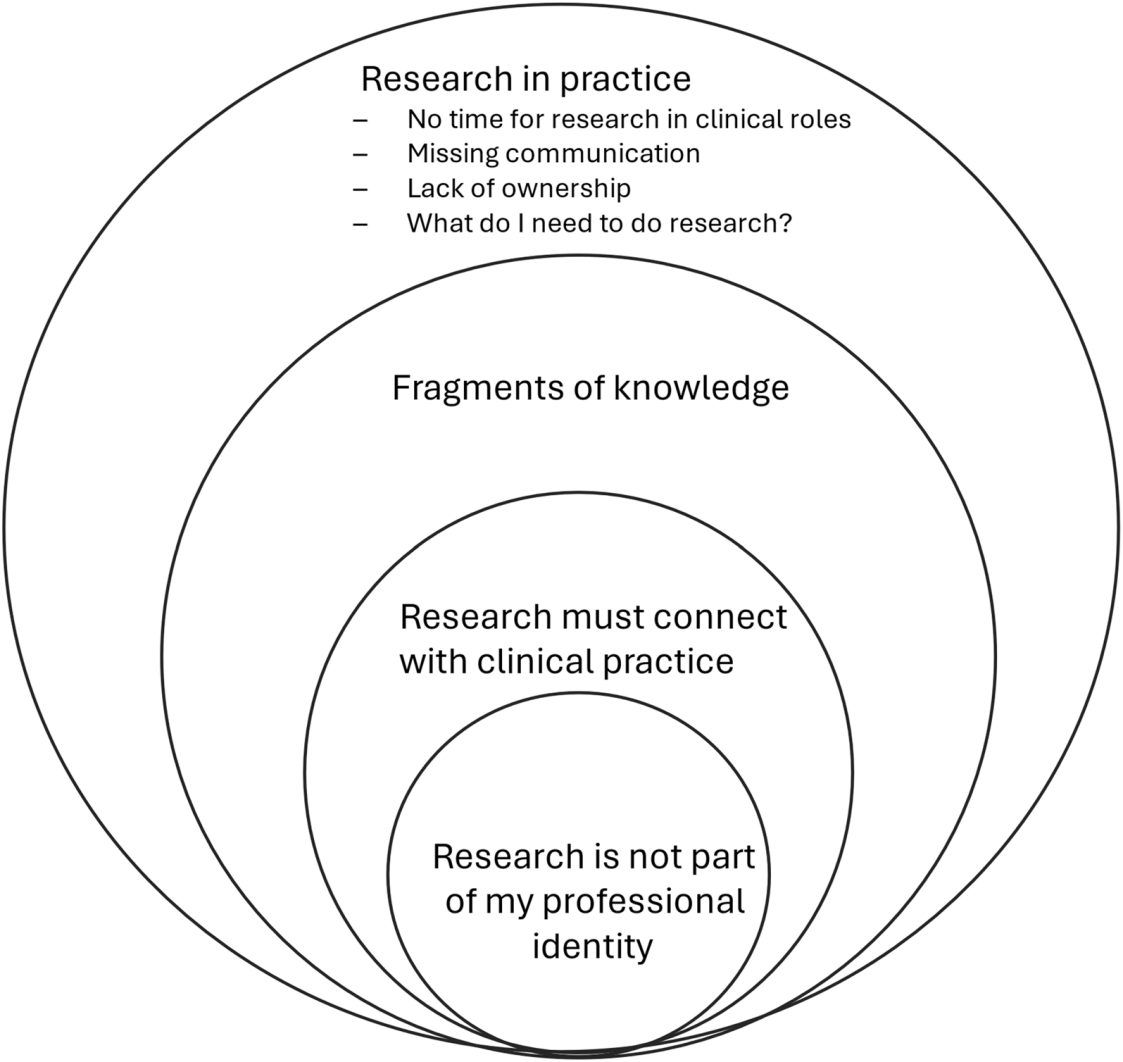
Study themes and subthemes

The following analysis represents analyses using both survey responses and interview data. The thematic analysis resulted in four major themes: research must connect with clinical practice; fragments of knowledge; research in practice; and research is not part of my professional identity. The theme, research in practice, comprised four subthemes: no time for research in clinical roles, missing communication, lack of ownership and what do I need to do research. Each of these themes and subthemes are described in more detail below. Quote attributions connected with survey responses begin with SP; quote attributions connected with interview responses begin with IP. See Fig. 1.

### Theme: research must connect with clinical practice

A core belief across participants was that research must connect with clinical practice. Participants perceived that research should be grounded in a focus on direct clinical care if it is to be both personally meaningful and valued at a systems level. There was a consistent view that the research must be relevant to improving clinical practice if it is to be justified in the context of minimal available time.

*Connecting to people’s everyday practice I think is important* (IP-13).

*I think it’s kind of hard because a lot of this evidence we see on the ground, it’s not really consolidated or there is* [sic] *no mechanisms – the mechanisms are lacking I think to translate that into hard data that we can push for more funds or whatever it might be* (IP-08).

Participants expressed a desire to improve the evidence base of care and use data to support the positive anecdotal outcomes they witness in their work as clinicians. Indeed, they viewed research as an ethical responsibility to measure the impact of care, shape practice and improve care. Research was considered a critical element in the evidence-based practice cycle.

*It really helps for me to be able to see that direct link* [between research and practice discussed during a recent seminar that the participant attended] *and just go, oh, that was really useful. Now I get it, now I know why I’m doing that* (IP-14).

*I’d like to research the efficacy of a mental health intervention, so it informs clinical practice* (SP-43).

Perhaps unsurprisingly then, their research interests were strong and diverse but always strongly linked to their clinical practice and/or service provision.

*I was interested in apathy in older adults* (IP-11).

*I’m also passionate about homelessness and mental health* (IP-07).

*The research I would be interested in is does running that program reduce the number of presentations to acute mental health services* (IP-15).

*I have a broad range of interests esp* [sic] *in using apps and smart phones to assist people with schizophrenia to organize their activities* (SP-26).

Not everyone expressed a desire to conduct the research themselves, nor do they need to; but it was clear that some participants did, as they told of a desire to seek research-related opportunities in the healthcare system.

*Yeah, I’ve always looked for the opportunities…probably I’m always thinking about my work and in the context of the opportunities of doing research and the evaluation* (IP-13).

Participants also spoke about enjoying a challenge and a love of learning, which, arguably, are essential ingredients for a willing clinician researcher.

*I’m a person who reads research. I have a natural inclination for it* (IP-15).

A few participants even expressed a desire to return to university to further progress their training in research. This part of their discourse was often followed with an acknowledgement of the large commitment required, the need to balance post-graduate university with other life responsibilities and of their decision that this aspiration needed to be postponed.

*I’d be interested in – I’ve wanted to do a PhD and I just put it off* (IP-02).

*I am interested, however, find balancing the research and clinical work difficult as research work requires a different kind of focus, a quiet space and extended periods of time to work through things* (SP-44).

### Theme: fragments of knowledge

Participants had been trained to conduct research as part of their studies at university. For most participants this training occurred as part of their undergraduate studies or qualifying degree for social work or occupational therapy. Participants referenced university placements that involved project-based work when reflecting on their research-related experiences. A few participants had further pursued post-graduate Master’s level study that was usually coursework-based, and some had worked previously as research assistants or in specific project roles. University appeared to be the main formal source of training for participants’ knowledge of research. For many this was some time ago.

*I did my Master’s a long time ago, so it was 2002* (IP-01).

*I think I’m just out of practice, its quite a long time ago…I would say I’d feel almost like I was starting from scratch if I was to tackle things like that again (*IP–05).

Practical, experientially acquired knowledge was discussed with reference to projects, quality improvement activities, evaluation and research conducted within clinical practice. Collectively, participants had experience in a variety of research-related tasks such as grant applications, literature reviews, ethics applications, co-design, recruitment, data collection (surveys, audits), quantitative analysis (statistics), qualitative analysis, mixed method analysis, presentations and writing for publication. However, individually, participants’ experience was typically connected to a few discrete research activities (e.g. grant applications and qualitative analysis), rather than experience across multiple tasks involved in completing a research project from start to end.

*A little bit of evaluation work, back in my community outreach days* (IP-06).

*I got all the research proposal put together. I got it past ethics* (IP-15).

Participants were conscious of their limited research-related experience in clinical practice, discussing how small and disparate – some using the term “bits and pieces” – their experience was. Participants spoke of having forgotten the extent of their research-related practices/training and at times seemed to be self-deprecating about the value of their research-related experiences. This sense of “bits and pieces” seemed to perhaps undermine participants’ sense of confidence and also their ability to identify as a clinician researcher.

*Research is not something I have led, but I’ve been involved in bits and pieces of things that have happened* (IP-01).

*I don’t know I mean I have done bits. I mean there’s always bits of research in training and I’ve done bits and pieces here and there* (IP-02).

*There’s kind of disparate sort of research that I know has gone on,* [which] *I’ve kind of dipped my toe in and supported* (IP-12).

This seems to be born out in their understanding about what research actually was. Roughly a third of the survey participants either did not know or only gave vague responses to the “what is the difference, if any, between project work, quality assurance, evaluation and research” question.

*They are all different (SP-47).*

*Difference is the stage where they take place?* (SP-16).

*It is all research, [it is] too difficult to answer* (SP-25).

Many of the survey participants tried to be thorough, providing thoughtful descriptions. Some definitions focussed on the differing purposes of the activities.

*Project work is developing and rolling out a project. QA – evaluating the efficacy of something. Evaluation – broad overview of if something works or not, taking on feedback, changing a project, and research academic investigation into a topic* (SP-49).

*They all serve a differing purpose. Project work is for creating. Quality assurance is to assess the quality of something. Evaluation is feedback. Research is gathering information on evidence base* (SP-140).

Some definitions focussed more on methods or processes.

*Different aspects of a whole process around research/projects. PW – working out how project/initiatives will be implemented and key components of a project. QA – measures to be implemented that ensure consistent quality in line with ethics, professional standards and legal guidelines. E – process of using different tools to measure outcomes of an intervention/project. R – inquiry into a determined area/topic. Process of finding outcomes to a question* (SP-14).

Some clinicians also used ethics to discriminate between the types, though their beliefs about ethics were not correct. Specifically, it does not matter whether an activity is called QA or internal review of programs/services or something else, what is more important is what the activity will be doing – it may still need ethical approval.

*Project work – small scale specific pieces of work. Usually involves consultation of some kind but doesn’t need formal questions or protocols. QA – internal review of programs/services offered. Good to get ethics approval but doesn’t need it. Evaluation – can be as simple as a survey to ask for feedback re training attended or more formal asking participants of a service about their outcomes. Research – ethics approved, formal protocol* (SP-134).

As an aside, the NHMRC advise that the primary purpose of the activity is less important, that is, whether it is called evaluation, quality assurance or research. Rather, the key considerations on which to base decisions on the need for an ethical review or not is whether the people involved (e.g. participants, members of the public or staff) will be exposed to any risk, burden, inconvenience or possible breach of privacy. In addition, in instances where a formal ethical application submission is not required, then oversight by others who are cognisant to ethically related issues (e.g. informed consent, legislative requirements, privacy, national/professional standards, etc.) is still necessary to ensure that ethical conduct is maintained during the given activity [4, 34].

### Theme: research in practice

This theme comprised four subthemes: no time for research in clinical roles, missing communication, lack of ownership and what I need to do research.

#### Subtheme: no time for research in clinical roles

Participants were explicit that their current clinical roles did not allow them time to conduct research; research was seen as “*a privileged thing to do*” (IP-01). A lack of time to conduct research seemed to be connected with a lack of opportunities to develop knowledge and skills to conduct research following teachings at university.

*[R]esearch, it’s like this one more thing that you’re just going to have to try and squeeze in. I’m like, I can’t, though. I haven’t got any more room* (IP-14).

The nature of the clinical work and the social environment seemed to place low expectations on research-related tasks being conducted as part of clinical work. As one participant stated “*well, there is just no capacity*” (IP-16). Some participants believed if they had further time they would conduct research , for example, “*well, if I had time, I’d do it*” (IP-16). Participants perceived a lack of time in clinical roles as negatively affecting their ability to have the “headspace” to think about research and plan and develop quality proposals.

*I think in the sense, just the nature of the work, we sort of have to prioritize the kind of clinical stuff* (IP-05).

*I think people just talk about not having the head space to even think more systematically* (IP-01).

Limited time interfered with the pursuit of creativity, rigour and innovation. They told of having no clear pathway to follow, being “shut down” or being forced to work outside of paid work hours – research as an extracurricular activity, if you like: “*No dedicated time to do research activities has to be done in own time*” (SP-25). One participant spoke of the loss of research skills given such limited time for doing research in clinical roles “*I think you lose that when you haven’t done it in a long time*” (IP-10), but most participants spoke of a lack of time with even getting to the start line.

### Subtheme: missing communication

A consistent message from participants was the lack of research-related communication about current and past projects that existed in the healthcare setting and even between each other. They were shrewdly aware there was not enough communication about research opportunities, processes and outcomes. As one participant stated, “*yeah, one of the things we’re aware is we don’t even know amongst ourselves* [own discipline] *who’s doing what*” (IP-01).

*That’s my impression. I just don’t hear people talk about research projects or seem to be seeing a lot happening in regards to that* (IP-15).

*So that still continues, but I’m not clear where that’s up to. I have no idea what the outcome or what the results have been…I know there’s research going on, I’m just not – it’s not visible to me as much as what I would have hoped* (IP-12).

Participants perceived communication about research was not part of the usual dialogue of clinicians and that if you wanted to follow up on research that had occurred, you need to proactively contact and “chase” people for such information – a great deal of work in a time-pressured environment.

*But again, we didn’t get shared the – I had to chase up to find out what the outcome of that research was so that we could get a copy of it to find out what happened. But again, I think the communication around once research is complete or parts of it are complete, letting people know that that’s being done is probably something that I think would be really, really helpful* (IP-01).

Communication was viewed as promoting the visibility of research and related opportunities. One participant provided a positive example of information sharing about research amongst peers yet concluded that this was an exception to the norm.

[W]*e are getting better at sharing that information to say well, you know this is what we’ve collected and this is how we’ve shaped the group program to better meet the needs…but again that’s kind of more the exception I think than the norm* (IP-03).

### Subtheme: lack of ownership

Most participants reported they had not led a research project and they had no experience in owning a research project from conception to completion. For some participants the experience of completing research had involved a lack of clarity around ownership of the work and this had been a confusing and undermining aspect of the experience, particularly when participants did not feel skilled to lead a research project.

*So when I was working with them, I felt they were really driving it and I was really just assisting* (IP-02).

Some participants seemed uncertain about whether they even wanted leadership experience in running a research project. Participants considered several negative consequences of this lack of ownership of research projects in the clinical environment that they had observed including a lack of shared vision and the project being disbanded.

*They’re all so busy, that group just fell away because no one wanted to even lead it* (IP-01).

*It’s not driven by anybody. It’s promoted by everyone, but it’s not – there is no shared vision* (IP-16).

### Subtheme: what I need to do research

Many participants were explicit about their lack of awareness of resources, support and opportunities for research in the health system in which they worked as clinicians.

*I guess my point of doing this* (the interview) *was to say that I don’t know much about what’s there, and I wonder if there aren’t that many opportunities available to – or we don’t know enough about what’s available to us* (IP-04).

*I don’t even know where to begin to start talking to someone about these things* (IP-15).

[Available resources –] *None in a work context* (SP-19).

Others were able to suggest some people or resources that could support research-related pursuits.

*I know that there’s all systems at the library. I think there’s probably some – there’s probably classes. There’s probably library training around all of that but, yeah, I don’t actually* [know] (IP-02).

*Yeah, so obviously the research OT lead would be the first point of call* (IP-12).

However, what was striking was that very few had actually used these resources, and no one expressed any intent for the same. “Someone to talk to” seemed to be highly valued, however, there seemed no intent to act in the near future. Some wondered whether they needed further training to conduct research but did not express any plans for action such as booking into the training offered through the hospital library – despite the interest noted previously.

### Theme: research is not part of my professional identity

While most participants expressed an interest in research and could name clinically relevant fields of study strongly connected to their clinical practice and/or service provision, doubt was expressed about their “natural” ability as a researcher. They reflected on their “rusty” skills and some believed they were not “technologically savvy”. Some felt intimidated by research and perceived they would be “floundering” with the process.

*I’m not naturally predisposed to do this* [research] *and I hadn’t prepared or planned for it…I don’t feel naturally academic* (IP-08).

*Thinking about it* [research] *overwhelms me* (SP-09).

One participant likened research-related tasks to an “indulgent” activity in a busy, time-pressured clinical environment; time pressures to conduct research also seemed to decrease participants’ confidence and ability. A couple of participants spoke of their preference to perform clinical work rather than research-related tasks. One participant questioned whether such negative perceptions of research as “scary” was due to a lack of exposure to research within the clinical environment and role.

*You then go into a clinical role, which I did straight away, and have always worked in clinical roles. You don’t really get exposed to much of it* [research]. *It can become quite scary and overwhelming* (IP-04).

*I work with a lot of our new graduates from all of the universities, and they always just impress me and make me want to run and hide in a corner, because they’re all so damn smart* (IP-01).

Participants in this study were skilled clinicians. Yet the undercurrent in these narratives was that research was not part of their identity. This was evident in their beliefs about themselves in their current allied health role.

*I don’t think of myself as a researcher…we don’t think about our day-to-day practices involving those things* (P01)

*We don’t think in the research mindset – it’s all clinical* (IP-12).

*I think I’m 4 years into the career and it has basically been clinical casework* (IP-08).

Additionally, it was evident in the embedded messages they received from the organization, that is, that research was not a priority or focus. Indeed, for the most part, research was not on the organizational radar at all thus shaping the context within which the clinicians worked.

*Sometimes I feel like I have good ideas but because of the pressures of seeing consumers that always gets pushed to the side* (IP-11).

*In clinical mental health…team leaders do not talk about this stuff* [research] *to everybody. Management doesn’t talk about this stuff to everybody. It’s left to supervision within their clinical area and that is too broad…there’s nobody driving it…there’s no shared vision* (IP-16).

*Any kind of research! We just don’t focus on that kind of thing here enough. It’s very much about working clinically. That’s the focus* (SP-44).

## Discussion

Arguably the most interesting and suggestive finding in our study points to a gap that, unless directly addressed, will likely thwart the longer-term success of research training programs. This was the observed disconnect between, on the one hand, clinicians being interested in doing research and able to name a range of salient practice-based research topics, and on the other hand, clearly communicating how their professional identity did not include research-related activities. Of course, not every participant expressed a desire to be a clinician researcher nor should they, and nor did every participant volunteer a research topic, but many did.

Participants in this study were skilled clinicians whose remit often included advocating on behalf of consumers and their families for whom they have been charged with care. Interest in research linked to their practice was evident in their discourses. Their roles require good problem-solving skills. They were able to nominate a range of useful ways to progress practice-based research within their organization. They could articulate ways to advance their own research-related knowledge and skills. Yet, they seemed to lack agency to implement such solutions to change their current situation. The undercurrent in these narratives was that research was not part of their professional identity. This was evident in their beliefs about themselves that seemed to be both explicitly and implicitly reinforced by the organizational culture.

Motivators and barriers to research were not the key focus of this study, however, perhaps unsurprisingly, they were prominent in the narratives in this study and were largely those already reported in the literature [e.g. 10, 11, 13, 14, 16]; especially as it is well accepted in many quarters that public mental health in Australia is severely underfunded and is a sector in need of reform [26]. Expanding clinical roles to include research with a clear focus on improving clinical care were strong motivators evident in our study and common in the studies based in general medical settings [10–16]. There was an even stronger overlap in barriers to research that included lack of skills, perceived (over)complexity of research, other work priorities and resource deficiencies that included lack of time, funding, suitable backfill, administrative support and technical expertise evident in studies set in general medical settings [10–18, 20–22] as well as in the current study. This current research study extends the literature to dedicated public mental health settings, noting the lack of time and lack of organizational support featuring quite strongly.

Allied health professionals have lamented the lack of research capacity in the workforce for decades. For example, in 1994, Selker [35] advised improvement in clinical research training, mentoring and funding was necessary to increase allied health professionals’ clinical research output. The body of work discussing research-building frameworks for allied health professionals has been comprehensive enough for a systematic review in 2018 [36]. The review outcome was the development of a targeted research capacity-building framework and recommendations that an integrated “whole of system” approach, including all levels of leadership and management, would be necessary for implementation success.

Research capacity-building strategies proposed by others over the years include target clinicians who are already interested in research [14, 16, 18] and focus on supporting team leaders and managers who are research literate and/or embedding research clinicians within teams [10, 16, 18]. Improved leadership and removal of bureaucratic constraints need to be achieved [37]. Provision of feedback on the progress and outcomes of the implemented innovation have also been recommended. The discourse of our study highlights the need to foster good teamwork and increased communication across all levels of the organization and can be directly linked to these recommendations. The literature also advises that training needs to have specific tangible targets rather than be generalist skills-based programs, needs to be ongoing rather than time-limited, conducted in situ (i.e. in the workplace) and preferably involving whole teams rather than scattered individuals and with the ready availability of mentors (preferably in-house) [38–43]. “Slack resources”, that is, spare resources/capacity, are required to foster innovative practice [44, 45] and equally research, which is consistent with our findings. We also support the implementation of these strategies.

Nonetheless, the research capacity and culture of health organizations, health service teams and individual health professionals has remained within the low and moderate range across many Australian-based health service settings, and especially low in the public mental health setting within which the current study was set [28]. This suggests that to date, other barriers had yet to be identified. Our results suggest this gap in our understanding is that research is not perceived to be part of the allied health professional identity. To that end, we recommend the integration of research-related skills into allied health professionals’ identity. We suggest that discipline educators pre-graduation (in university) and post-graduation (in-house educators) should view research-related activities as an everyday part of clinical roles, especially if we think of evidence-based practice requiring similar skills to clinical research [46]. Arguably, a shift in discipline education towards the clinician scientist modus operandi [47], that is, including a stronger focus on research-related skills from the beginning of undergraduate education, is required, including making explicit the research-related skills the students already assimilate during their coursework [48]. Finally, we would add that updating position descriptions and role responsibilities to include research-related skills and activities that enable clinicians to understand, appraise and translate research into clinical practice.

To be clear, we are not suggesting that every allied health clinician needs to be a clinician–researcher, but they all need a reasonable level of research-related skills and/or competencies to appraise, practice, evaluate and adapt their evidence-based practice [49]. Without the adoption of each and all of the above recommendations, it is difficult to see how the recent Royal Commission’s advocacy for stronger multidisciplinary translational research and widespread evaluation will be realized here in Victoria Australia.

In 2019, the Victorian government established a Royal Commission to undertake a 24-month inquiry into Victoria’s mental health system following widespread acknowledgement of the current broken system that had failed to meet people’s needs. Individuals with lived experience and their families, carers, supporters, mental health workers, researchers and service providers were consulted. Indigenous people, LGBTIQ+ people and people with different cultural backgrounds were also consulted. In short, 65 comprehensive recommendations were made, which included: recommendation 23, which called for multidisciplinary and translational trauma research; recommendation 36, which called for dedicated research into mental illness and substance use or addition; and recommendation 63, which called for facilitation of translational research throughout the mental health and wellbeing system. The full report is available at Royal Commission into Victoria’s Mental Health System—final report | vic.gov.au (www.vic.gov.au).

Two strengths of this study are the focus on clinicians based within a dedicated Australian public mental health setting and the triangulation of data sources (survey and interview) that enhance the rigour and credibility in our results. Limitations of this study are that it focused on two disciplines only, that is, mental health social workers and occupational therapists, and was based in a single organization. Further insight would be gained by expanding this line of inquiry to include other disciplines in public mental health, as well as other public mental health organizations.

This study found that research and research-related activities were not considered part of the mental health social workers’ and occupational therapists’ professional identity. Dealing with this issue will be necessary to the realization of the emphasis on evidence-based practice within clinicians’ professional peak-body associations’ codes of practice and government mandated practice standards. Arguably, shifting mental health clinicians and their workplaces perceptions to accept that research is a part of their professional identity will be key to their ability to maintain their fledgling research-related skills post-graduation and developing those same skills once in the workforce. A concerted effort needs to be made across both tertiary education and the public mental health system so that both clinicians and services view research-related activities as an everyday part of clinical roles, especially if we think of evidence-based practice requiring similar skills as clinical research. Foundations for this bridge will need to be built into organization policy, culture and management structure, as well as clinicians’ knowledge, understanding and practice. We provided several strategies to help.

## Data Availability

The datasets used and analysed during the current study are available from the corresponding author on reasonable request.

## References

[1] Practice support: Professional practice documents and publications [https://otaus.com.au/practice-support/position-statements]. Accessed 19 Jan 2024.

[2] Practice Standards 2023 [https://www.aasw.asn.au/about-aasw/ethics-standards/practice-standards/]

[3] Code of conduct [https://www.ahpra.gov.au/Resources.aspx]. Accessed 19 Jan 2024.

[4] National Health and Medical Research Council, Australian Research Council, Australian Vice-Chancellors' Committee: National statement on ethical conduct in human research (updated 2018). NHMRC; 2007.

[5] Bordens KS, Abbott BB. Research design and methods: a process approach. 4th ed. California: Mayfield Publishing; 1999.

[6] Carter EJ, Mastro K, Vose C, Rivera R, Larson EL. Clarifying the conundrum: evidence-based practice, quality improvement, or research?: the clinical scholarship continuum. J Nurs Adm. 2017;47:266–70. 28422932 10.1097/NNA.0000000000000477

[7] Victorian allied health research framework [https://www.health.vic.gov.au/allied-health-workforce/allied-health-research]. Accessed 19 Jan 2024.

[8] Practice standards [https://www.aasw.asn.au/practice-standards-2023/] Accessed 19 Jan 2024.

[9] Evidence-based practice position statement [https://otaus.com.au/practice-support/position-statements]

[10] Borkowski D, McKinstry C, Cotchett M. Research culture in a regional allied health setting. Aust J Prim Health. 2017;23:300–6. 28377009 10.1071/PY16085

[11] Cordrey T, King E, Pilkington E, Gore K, Gustafson O. Exploring research capacity and culture of allied health professionals: a mixed methods evaluation. BMC Health Serv Res. 2022;22:85. 35039018 10.1186/s12913-022-07480-x PMC8764821

[12] Elphinston RA, Pager S. Untapped potential: psychologists leading research in clinical practice. Aust Psychol. 2015;50:115–21. 10.1111/ap.12102

[13] Frakking T, Craswell A, Clayton A, Waugh J. Evaluation of research capacity and culture of health professionals working with women, children and families at an Australian public hospital: a cross sectional observational study. J Multidiscip Healthc. 2021;14:2755–66. 34629876 10.2147/JMDH.S330647 PMC8496547

[14] Matus J, Tearne JE, Blyth K, Coates S, Pearson S, Cavalheri V. An evaluation of research capacity and culture in a sample of Western Australian Allied Health professionals. Tasman Med J. 2021;3:23–9.

[15] Oliver-Baxter J, Brown L, McIntyre E. Surviving or thriving in the primary health care research workforce: the Australian experience. Aust J Prim Health. 2017;23:183–8. 27737728 10.1071/PY15190

[16] Pager S, Holden L, Golenko X. Motivators, enablers, and barriers to building allied health research capacity. J Multidiscip Healthc. 2012;5:53–9. 22396626 10.2147/JMDH.S27638 PMC3292402

[17] Pain T, Petersen M, Fernando M. Building allied health research capacity at a regional Australian hospital: a follow-up study. Int J Allied Health Sci Pract. 2018;16:1–10.

[18] Friesen EL, Comino EJ. Research culture and capacity in community health services: results of a structured survey of staff. Aust J Prim Health. 2017;23:123–31. 27531587 10.1071/PY15131

[19] Wenke R, Weir KA, Noble C, Mahoney J, Mickan S. Not enough time for research? Use of supported funding to promote allied health research activity. J Multidiscip Healthc. 2018;11:269–77. 29950853 10.2147/JMDH.S157034 PMC6016580

[20] Brandenburg C, Noble C, Wenke R, Hughes I, Barrett A, Wellwood J, Mickan S. Relationship between research culture and research activity of medical doctors: a survey and audit. J Multidiscip Healthc. 2021;14:2137–50. 34408428 10.2147/JMDH.S319191 PMC8364349

[21] Gill SD, Gwini SM, Otmar R, Lane SE, Quirk F, Fuscaldo G. Assessing research capacity in Victoria’s south-west health service providers. Aust J Rural Health. 2019;27:505–13. 31814198 10.1111/ajr.12558

[22] Wenke R, Noble C, Weir KA, Mickan S. What influences allied health clinician participation in research in the public hospital setting: a qualitative theory-informed approach. BMJ open 2020, 10:e036183-NA. 10.1136/bmjopen-2019-036183 PMC7443264 32819986

[23] Mental health: expenditure on mental health-related services [https://www.aihw.gov.au/mental-health/topic-areas/expenditure]. Accessed 24 Jan 2024.

[24] Australian Institute of Health and Welfare: mental health conditions and substance use disorders a leading cause of diseach burden in 2023. Canberra: AIHW; 2023.

[25] Yung AR, Milicevic M, Berk M. Fair funding for mental health research. Aust N Z J Psychiatry. 2023;57:1091–4. 37243396 10.1177/00048674231177226 PMC10363923

[26] Mental health [https://www.aihw.gov.au/mental-health/overview/australias-mental-health-services]

[27] Kothari CR: Research methodology: Methods and techniques. New Age International; 2004.

[28] Migliorini C, McDowell C, Turville M, Bevilacqua J, Harvey C. Research capacity and culture in an Australian metropolitan public mental health service: scoping the skills and experience of social workers and occupational therapists. BMC Med Educ. 2022;22:864. 36517812 10.1186/s12909-022-03936-0 PMC9749178

[29] Harris PA, Taylor R, Thielke R, Payne J, Gonzalez N, Conde JG. Research electronic data capture (REDCap)–A metadata-driven methodology and workflow process for providing translational research informatics support. J Biomed Inform. 2009;42:377–81. 18929686 10.1016/j.jbi.2008.08.010 PMC2700030

[30] LaDonna KA, Taylor T, Lingard L. Why open-ended survey questions are unlikely to support rigorous qualitative insights. Acad Med. 2018;93:347–9. 29215376 10.1097/ACM.0000000000002088

[31] Braun V, Clarke V: Successful qualitative research: a practical guide for beginners. sage; 2013.

[32] Patton MQ. Qualitative research and evaluation methods. 3rd ed. Thousand Oaks: Sage Publications; 2002.

[33] Carter N, Bryant-Lukosius D, DiCenso A, Blythe J, Neville AJ. The use of triangulation in qualitative research. Oncol Nurs Forum. 2014;41:545–7. 25158659 10.1188/14.ONF.545-547

[34] Ethical considerations in quality assurance and evaluation activities [https://www.nhmrc.gov.au/about-us/resources/ethical-considerations-quality-assurance-and-evaluation-activities]. Accessed 19 Jan 2024.

[35] Selker LG. Clinical Research in Allied Health. J Allied Health. 1994;23:201–28. 7721645

[36] Matus J, Walker A, Mickan S. Research capacity building frameworks for allied health professionals—A systematic review. BMC Health Serv Res. 2018;18:716. 30219065 10.1186/s12913-018-3518-7 PMC6139135

[37] Aarons GA, Sawitzky AC. Organizational culture and climate and mental health provider attitudes toward evidence-based practice. Psychol Serv. 2006;3:61–72. 17183411 10.1037/1541-1559.3.1.61 PMC1712666

[38] Brekke JS, Phillips ES, Pancake L, Lewis J, Duke J. Implementation practice and implementation research: a report from the field. Res Soc Work Pract. 2009;19:592–601. 10.1177/1049731509335561

[39] Corrigan PW, Boyle MG. What works for mental health system change: evolution or revolution? Adm Policy Ment Health. 2003;30:379–95. 12940682 10.1023/A:1024619913592

[40] Corrigan PW, McCracken SG, Blaser B. Disseminating evidence-based mental health practices. Evid Based Ment Health. 2003;6:4–5. 12588813 10.1136/ebmh.6.1.4

[41] Maynard BR. Social service organizations in the era of evidence-based practice: the learning organization as a guiding framework for bridging science to service. J Soc Work. 2009;10:301–16. 10.1177/1468017309342520

[42] Taylor NF, Harding K, Shields N. Stepping into research: instructor’s manual. Melbourne, Victoria: Eastern Health and La Trobe University; 2020.

[43] Taylor NF, Porter J, Lynch L, Horne-Thompson A, Wallis J, Kerridge G, Harding KE, Wilby A, Joy AM, Kaminski MR, et al. Evaluating the introduction of an allied health clinical research office at a health service: effects on research participation, interest, and experience of allied health professionals. J Allied Health. 2019;48:46–53. 30826830

[44] Titler MG: The evidence for evidence-based practice implementation. In Patient Safety and Quality: an evidence-based handbook for nurses. Edited by Hughes RG. Rockville (MD): Agency for Healthcare Research and Quality; 2008 21328752

[45] Lu W, Song X, Hou C, Zhu J. The effect of slack resources on innovation performance and the environmental adaptability of public hospitals: the empirical evidence from Beijing of China. Front Public Health. 2022. 10.3389/fpubh.2022.904984. 35844888 10.3389/fpubh.2022.904984 PMC9283980

[46] Harvey C, O’Hanlon B. Family psycho-education for people with schizophrenia and other psychotic disorders and their families. Aust N Z J Psychiatry. 2013;47:516–20. 23393269 10.1177/0004867413476754

[47] Weggemans M, Friesen F, Kluijtmans M, Prakken B, ten Cate O, Woods NN, Rosenblum N. Critical gaps in understanding the clinician-scientist workforce: results of an international expert meeting. Acad Med. 2019;94:1448–54. 31135403 10.1097/ACM.0000000000002802

[48] Vieno K, Rogers KA, Campbell N. Broadening the definition of ‘research skills’ to enhance students’ competence across undergraduate and master’s programs. Educ Sci. 2022;12:642. 10.3390/educsci12100642

[49] Dawes M, Summerskill W, Glasziou P, Cartabellotta A, Martin J, Hopayian K, Porzsolt F, Burls A, Osborne J. Second international conference of evidence-based health care teachers developers: sicily statement on evidence-based practice. BMC Med Educ. 2005. 10.1186/1472-6920-5-1. 15634359 10.1186/1472-6920-5-1 PMC544887

